# GABARAP activates ULK1 and traffics from the centrosome dependent on Golgi partners WAC and GOLGA2/GM130

**DOI:** 10.1080/15548627.2016.1159368

**Published:** 2016-03-17

**Authors:** Justin Joachim, Sharon A. Tooze

**Affiliations:** The Francis Crick Institute, Lincoln's Inn Fields Laboratories, London, UK

**Keywords:** Atg8, centrosome, GABARAP, GM130, GOLGA2, golgi, photoactivation, ULK1, WAC

## Abstract

WAC and GOLGA2/GM130 are 2 Golgi proteins that affect autophagy; however, their mechanism of action was unknown. We have shown that WAC binding to GOLGA2 at the Golgi displaces GABARAP from GOLGA2 to allow the maintenance of a nonlipidated centrosomal GABARAP pool. Centrosomal GABARAP can traffic to autophagic structures during starvation. In addition GABARAP specifically promotes ULK1 activation and this is independent of GABARAP lipidation but likely requires a LIR-mediated GABARAP-ULK1 interaction.

How the autophagosome formation machinery is regulated is not fully understood. In light of this, WAC (WW domain containing adaptor with coiled-coil) was identified by our group as a novel positive regulator of autophagosome formation. WAC localizes to the nucleus and the Golgi where it regulates epigenetics and post-mitotic Golgi reassembly, respectively. Both the WW and coiled-coil domains of WAC participate in protein-protein interactions. In search of a mechanism of WAC's effects on autophagy we performed immunoprecipitation and mass spectrometry to find novel WAC interaction partners. We found GOLGA2 as a new direct WAC interaction partner. Interestingly, GOLGA2 has previously been implicated as a negative regulator of autophagy; however, how this occurs was unknown. GOLGA2 is thought to be an elongated coiled-coil molecule and functions in multiple cellular processes at the Golgi, including vesicle tethering, Golgi structural maintenance and centrosome function.

WAC knockdown reduces autophagy flux as measured by MAP1LC3B lipidation, SQSTM1/p62 degradation and WIPI2B puncta formation. WAC is required for the maximal phosphorylation of ATG13 at serine 318 by the autophagy-initiating kinase ULK1. In contrast, GOLGA2 knockdown modestly increases autophagy flux. A 10 amino acid region in the C-terminal coiled-coil domain of WAC is required for interaction with the C-terminal region of GOLGA2. Moreover, WAC interaction with GOLGA2 is important for autophagosome formation.

The link between WAC-GOLGA2 and the autophagy machinery is through the Atg8 homolog GABARAP (Fig. 1). GOLGA2 binds GABARAP directly and with specificity, as other human Atg8s interact with GOLGA2 to a lesser extent. Interestingly, in vitro competition experiments with purified proteins, and knockdown experiments in HEK293A cells show that WAC competes for binding with GABARAP and drives it off GOLGA2. How this occurs and is regulated is unknown, but it could be through steric hindrance or a conformation change. WAC knockdown results in enhanced binding of GABARAP to GOLGA2 and accumulation of GABARAP at the Golgi. Surprisingly, we observed a pool of nonlipidated GABARAP localized to the pericentriolar material (PCM) of the centrosome. The PCM is a dynamic matrix of coiled-coil proteins, embedded in which are microtubule nucleating TUBG/γ-tubulin ring complexes and centrioles. WAC maintains the pool of GABARAP at the PCM by preventing GABARAP binding to the Golgi. This Golgi-centrosome crosstalk of GABARAP is mediated through microtubules, as depolymerization of the microtubule cytoskeleton results in a relocalization of GABARAP from the PCM to Golgi ministacks adjacent to ER-exit sites.

**Figure 1.**
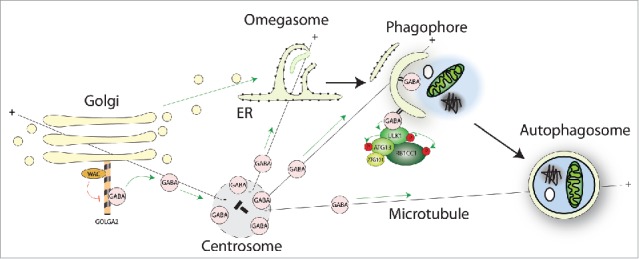
Through its C-terminal coiled-coil domain WAC interacts with GOLGA2 at the Golgi. The WAC-GOLGA2 interaction prevents excessive binding of GABARAP to GOLGA2 to allow the maintenance of a pool of GABARAP at the pericentriolar material of the centrosome. Golgi-to-centrosome transport of GABARAP likely requires microtubules. Centrosomal GABARAP can traffic to forming or possibly formed autophagosomes upon starvation, which may be directed by microtubules. GABARAP functions to scaffold the ULK complex and to promote its kinase activity during autophagosome biogenesis.

During starvation-induced autophagy, photoconversion experiments revealed that a PCM-localized pool of EosFP-GABARAP translocates to forming or formed autophagosomes. However, when GABARAP is bound to the Golgi after WAC depletion or microtubule depolymerization, it does not relocalize to autophagosomes. Thus, the Golgi-centrosome pool of GABARAP, controlled by WAC and GOLGA2, appears to regulate GABARAP delivery to autophagosomes.

The function of GABARAP at the Golgi or the centrosome remains unclear. GABARAP may have an autophagy-independent Golgi role related to vesicular trafficking. Several autophagy proteins, such as ATG16L1 and BECN1, are at the Golgi, raising the possibility that the Golgi is a signaling hub for autophagy regulation. Moreover, the role of GABARAP at the centrosome is not fully understood, nor is it known whether other autophagy proteins reside there. GABARAP may direct centrosome-regulated processes such as microtubule organization or cell division. In addition the relationship between centrosomal GABARAP and primary cilia-associated GABARAP is unclear. A number of autophagy proteins localize to the basal body (formed from the mother centriole) of the primary cilium upon serum starvation, a stimulus that drives ciliogenesis. However, some autophagy proteins, such as MAP1LC3B, are reported to be associated with the basal body in a serum-independent manner; GABARAP may be similar.

Surprisingly, knockdown of GABARAP, but not other Atg8s, reduces ULK1 activity and this connects the WAC-mediated activation of ULK1 with WAC's effects on GABARAP trafficking. Moreover, in the presence of nonlipidated GABARAP, but not MAP1LC3B, the LIR motif of ULK1 is required for its maximal kinase activity. Therefore, GABARAP likely enhances ULK1 activation through direct LIR-mediated binding downstream of regulation by WAC-GOLGA2. These data suggest that in addition to acting as an autophagy adaptor, GABARAP could function to enforce ULK1 activity during autophagosome biogenesis.

Several pieces of data suggest that ULK1 affects autophagy at post-initiation stages. PtdIns3P, thought to be downstream of ULK1, is required to maintain ULK1 at the omegasome. ULK1 is partially recruited to the autophagosome through binding to Atg8s using its LIR motif, and mutation of the ULK1 LIR motif causes accumulation of early autophagic structures. Finally, a ULK1 inhibitor accumulates stalled early autophagic structures. These data are reminiscent of knockdown of the GABARAPs, which also accumulates early autophagic structures. A clue to an intimate relationship between GABARAP and ULK1 is that ULK1 preferentially binds the GABARAPs over the LC3s: GABARAP can recruit and scaffold the ULK complex onto the phagophore. The reason for this preferential binding is unknown. However, our data suggest the GABARAP-ULK1 interaction specifically primes the activity of the kinase and thus may explain a preference for ULK1-GABARAP interactions.

## Conclusion

The divergence of the Atg8 homologs and the abundance of specific and common interactors raise fascinating questions: What is the extent of redundancy or specialization of the Atg8s? What is the purpose of numerous LIR-dependent interactions and how are these regulated? Recent studies of specific Atg8 family members are starting to yield some answers. An emerging theme in autophagy research is the idea of autophagy as an integrated complex network, in contrast to a linear pathway. Recent research has elucidated feedback/feedforward signaling loops, but also demonstrated that ATG proteins can have multiple functions, directed by discrete binding partners at the initiation/nucleation and maturation stages of autophagosome biogenesis.

